# Post-weight loss changes in fasting appetite- and energy balance-related hormone concentrations and the effect of the macronutrient content of a weight maintenance diet: a randomised controlled trial

**DOI:** 10.1007/s00394-020-02438-3

**Published:** 2020-12-02

**Authors:** Mari Näätänen, Marjukka Kolehmainen, David E. Laaksonen, Karl-Heinz Herzig, Kaisa Poutanen, Leila Karhunen

**Affiliations:** 1 Department of Clinical Nutrition, Institute of Public Health and Clinical Nutrition, University of Eastern Finland, P.O. Box 1627, 70211 Kuopio, Finland; 2 Department of Physiology, Institute of Biomedicine, University of Eastern Finland, Kuopio, Finland; 3 Internal Medicine, Institute of Clinical Medicine, Kuopio University Hospital, Kuopio, Finland; 4 Institute of Biomedicine, Medical Research Center (MRC), University of Oulu, University Hospital, Oulu, Finland; 5 Department of Gastroenterology and Metabolism, Poznan University of Medical Sciences, Poznan, Poland; 6 VTT Technical Research Centre of Finland, Espoo, Finland

**Keywords:** Macronutrients, Weight maintenance, Ghrelin, Leptin, Insulin, Peptide YY

## Abstract

**Purpose:**

We investigated the effects of the macronutrient composition of diets with differing satiety values on fasting appetite-related hormone concentrations after weight loss and examined whether the hormone secretion adapted to changes in body fat mass (FM) and fat-free mass (FFM) during the weight maintenance period (WM).

**Methods:**

Eighty-two men and women with obesity underwent a 7-week very-low-energy diet (VLED) and were then randomised to a higher-satiety food (HSF) group or a lower-satiety food (LSF) group during 24-weeks of the WM. The groups consumed isoenergetic foods with different satiety ratings and macronutrient compositions.

**Results:**

During the WM, the HSF group consumed more protein and dietary fibre and less fat than the LSF group, but the groups showed similar changes in body weight and fasting appetite-related hormones. In the whole study sample, VLED induced 12 kg (*p* < 0.001) weight loss. At the end of the WM, weight regain was 1.3 kg (*p* = 0.004), ghrelin concentration increased, whereas leptin, insulin, and glucose concentrations decreased compared to pre-VLED levels (*p* < 0.001 for all). Peptide YY did not differ from pre-VLED levels. Changes in ghrelin levels were inversely associated with changes in FFM during weeks 0–12 of the WM (*p* = 0.002), while changes in leptin and insulin levels were positively associated with changes in FM during weeks 0–12 (*p* = 0.015 and *p* = 0.038, respectively) and weeks 12–24 (*p* < 0.001 and *p* = 0.022) of the WM.

**Conclusions:**

The macronutrient composition of an isoenergetic WM diet did not affect fasting appetite-related hormone concentrations. Leptin and insulin adjusted to the reduced FM, whereas ghrelin reflected FFM during the first months of the WM.

**Trial registration:**

isrctn.com, ID 67529475.

## Introduction

According to the dynamic weight set point theory, the normal range of biological weight is elevated in people with obesity [1]. Consequently, the basal levels of appetite-related hormones that reflect body mass, e.g. ghrelin, leptin, and insulin, are altered in people with obesity compared to those of people with normal body weights [2]. Reducing weight by dietary restriction results in counter-adaptive changes in physiological mechanisms involved in the regulation of appetite and energy balance [3]. These changes include an increase in the fasting concentration of orexigenic hormone ghrelin and a decrease in the fasting concentrations of anorexigenic hormones such as leptin, insulin, and peptide YY (PYY) [4–8]. Functionally, weight loss-induced alterations in the regulatory mechanisms have been related to increased feelings of hunger and decreased energy expenditure in some studies [7, 9]. Thus, these physiological changes may be involved in constituting an energy gap in which the body's energy requirements are lower than the hunger drive [3]. Ultimately, the physiological hunger gap accompanied by behavioural factors may result in difficulty to maintain the reduced weight. Moreover, the weight loss-induced decrease in leptin and insulin levels has been found to be greater than would be expected on the basis of the reduced body fat mass (FM) [10]. Thus, the negative energy balance and the loss of body mass could disrupt hormone secretion and unbalance its relation to the body’s energy reserves.

It is evident that novel ways are needed to promote the normalisation of post-weight loss hormone secretion to facilitate overcoming the energy gap. High-protein [11] and high-fibre [3] diets have been associated with decreased hunger and successful weight management. The satiating effect of foods may vary depending on their macronutrient composition [12]. Only a few studies have investigated the effect of the macronutrient composition of a weight maintenance diet on appetite-related hormone secretion after weight loss [13–17]. It is still unclear whether the satiating value and thereby the macronutrient composition of a weight maintenance diet affect basal post-weight loss appetite hormone concentrations during weight maintenance.

At present, little is known about the persistence of changes in basal appetite-related hormone concentrations after weight loss. The hormonal changes have persisted over a 6-month [5] and a 12-month [7] weight maintenance period (WM) despite weight regain; accompanied  by a persistent increase in subjective evaluations of hunger and desire to eat [7]. Other studies have found that some hormones revert to pre-weight loss levels despite no weight regain occurring during a 6-month [18–20] and a 12-month [8] WM. Functionally, changes in leptin levels have been associated with changes in body weight or BMI during weight loss or WM [7, 18–20]. In contrast, the relationship between other peptide hormones and body weight or composition during post-weight loss WM is not as well known. Moreover, previous studies have investigated appetite-related hormone concentrations only before or after WM. Instead, the dynamic changes in hormone concentrations during weight maintenance are unclear.

We have reported earlier that two groups adhering to isoenergetic diets containing either higher or lower predetermined satiety value foods that differed in their macronutrient contents achieved equal success in maintaining weight reduction after very-low-energy diet (VLED) [21]. This success was accomplished with only a slight regain in the mean body weight during the WM (in average + 1.3% of the  body weight at the beginning of the WM). Nonetheless, the question remains whether the altered appetite-related hormone secretion after weight loss could adjust to the reduced energy reserves and body mass during the WM and reflect the changes in them, i.e. this would represent a normalisation of hormone secretion in people with obesity who succeeded in weight management.

To this end, we conducted a secondary analysis of our previous study [21]. The primary aim of this study was to investigate whether isoenergetic diets with differing macronutrient contents and with similar effects on body weight would affect fasting appetite-related hormone concentrations differently during a 24-week WM after weight loss. The secondary aim was to investigate whether the changes in post-weight loss hormone concentrations were associated with changes in body composition occurring during a 24-week WM, thus adopting to the reduced weight. We hypothesised that the WM diet containing higher-satiety foods (HSF) would attenuate the weight loss-induced counter-regulatory changes in fasting appetite-related hormone concentrations as compared to the WM diet containing lower-satiety foods (LSF). In addition, the associations between changes in fasting hormone concentrations and body composition could vary during the different phases of the WM, i.e. (1) early phase (weeks 0–12 of the WM) and (2) late phase (weeks 12–24 of the WM).

## Methods

### Participants

**Fig. 1:**
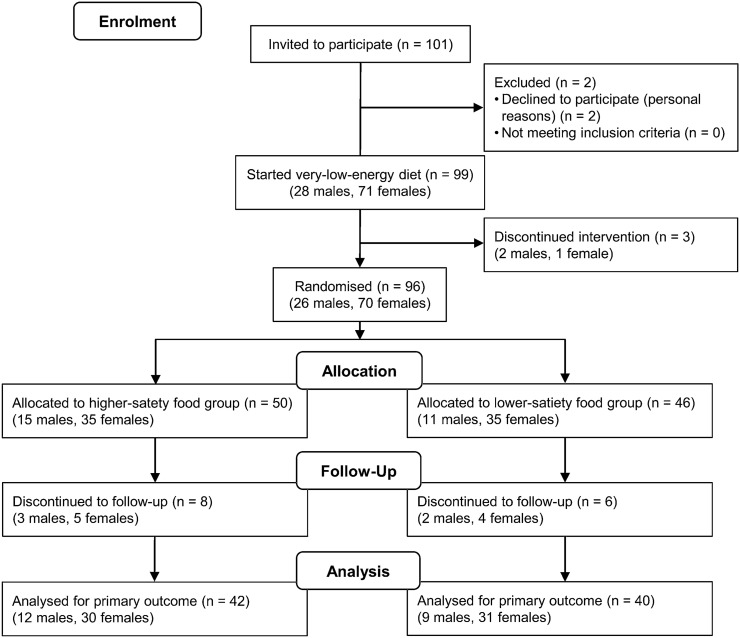
Participant flow chart

Participants were recruited through local newspaper advertisements and among individuals who had previously participated in studies conducted in the University of Eastern Finland. Eligibility criteria were body mass index (BMI) of 30–40 kg/m^2^ and age of 30–65 years. Exclusion criteria included BMI > 40 or < 30 kg/m^2^, type 1 or 2 diabetes, pregnancy, kidney or thyroid dysfunction, heart  or liver disease, polycystic ovary syndrome, diagnosed eating disorder, alcohol consumption > 16 (women) or > 24 (men) portions/week (1 portion equals 12 g of pure alcohol), neuroleptic or oral cortisone medication or any other diseases or medications that could prevent the participants from completing the study. A total of 101 participants were recruited; of those, 99 participants started the study (Fig. 1).

### Study design

The present study is a secondary analysis of a parallel arm randomised controlled trial. The study protocol has been described in detail previously [21]. In brief, all the participants underwent a 7-week weight-loss period (WL) with VLED products (Nutrifast, Leiras Finland Ltd) providing 600 kcal/day. Low-energy vegetables and energy-free beverages could be consumed ad libitum*.* After a subsequent 2-week transition phase of VLED, the participants were stratified by age and sex and randomly assigned to a HSF group or a LSF group for a 24-week WM. The allocation was made by the research group leader using a stratified blocked randomisation method with block sizes of 4 at a 1:1 ratio. The participants were numbered sequentially upon enrolment by the study technician conducting the recruitment, which determined the order in which the participants were randomised. The HSF group consumed intervention foods with a higher predetermined satiety value, and the LSF group consumed intervention foods with a lower predetermined satiety value as a part of their WM diet. The participants were not informed about the different satiety values of the intervention foods. They were advised to maintain the weight loss achieved during the WL without further weight reduction. In addition, the participants were instructed to keep their physical activity at their habitual level throughout the whole study.

The trial is registered at isrctn.com with the identifier 67529475.

### Weight maintenance diets

The satiety values of the intervention foods were predetermined in a separate test in controlled laboratory settings before the intervention, as described in detail previously [21]. The HSF products had higher protein and dietary fibre and lower fat content when compared to those of the LSF products. The intervention foods represented five food categories with every HSF product with a corresponding isoenergetic LSF product (Table 1). The participants received the intervention foods every two weeks from the study clinic at the University of Eastern Finland.

**Table 1:** Intervention foods in the study groups, number of portions instructed to be consumed daily, and the energy and macronutrient composition of one portion of the product

	Dairy		Bread			Cheese	Cold cuts	Vegetable patty
	Yoghurt 1	Yoghurt 2	Soft bread 1	Crisp bread 1	Soft/crisp bread 2			
Higher-satiety food group								
Portion size (g)	150.0	150.0	43.0	12.9	30.0	125.0	6.2	60.0
Portions/day^a^	1–2	1–2	1–3	1–3	1–3	0.25–0.75	4–10	1–2
Energy (kcal)	90.0	90.0	86.0	41.0	75.0	238.0	7.5	96.0
Carbohydrates (g)	6.0	6.0	16.3	7.7	14.0	1.9	0.1	5.0
Protein (g)	16.5	16.5	3.1	1.4	2.4	42.5	1.4	5.3
Fat (g)	0.3	0.3	0.6	0.3	0.6	6.3	0.2	6.2
Dietary fibre (g)	3.0	3.0	4.7	2.2	4.5	0	0.2	4.0
Lower-satiety food group								
Portion size (g)	130.0	110.0	25.0	12.5	6.3	19.0	13.4	60.0
Portions/day^a^	1–2	1–2	1–4	1–2	2–10	1–3	1–5	1–2
Energy (kcal)	91.0	91.0	70.0	50.0	20.0	55.0	20.0	105.0
Carbohydrates (g)	13.0	14.3	11.8	8.8	4.0	0.3	1.1	6.5
Protein (g)	4.7	3.9	2.2	1.3	0.7	3.8	1.2	2.8
Fat (g)	2.6	2.2	1.2	1.0	0.7	4.2	1.2	7.4
Dietary fibre (g)	0	0	0.8	0.8	1.2	0	0.4	2.0

^a^The number of portions consumed varied on a day to day basis and according to individually estimated energy content of diet

The energy content of the WM diet was determined individually for every participant according to calculated energy requirements as follows: (formula of Mifflin St-Jeor—5% to consider the likely reduction in basal metabolic rate due to weight loss) × 1.3 (physical activity level) − 333.45 kcal (to consider our previous experiences in a pilot study of a difference between reported energy intake and calculated energy requirements [21]). The number of intervention foods to be consumed daily was determined by the individual energy level with the intervention foods accounting for about 30% of the calculated total energy requirements. Otherwise, the diet consisted of freely selected foods. The participants were given written instructions about the amount of intervention foods to be consumed daily and a recommendation of the amounts of freely selected foods to be consumed from different food groups (vegetables, fruits and berries; dairy; potato and cereals; meat, poultry and fish; fats) to meet their individual daily energy requirements. However, the participants in both study groups were informed that, apart from the intervention foods, they could eat the freely selected foods ad libitum according to subjective sensations of hunger and satiety. During the whole study, the participants attended seven group sessions led by a dietician in which they received dietary counselling on the principles of VLED and weight management. Five sessions occurred during the WL, one at the beginning of the transition phase, and one at the beginning of the WM. Otherwise, during the WM, the participants received no dietary counselling apart from the written instructions described above.

To enhance adherence, the intervention foods were varied to some extent every two weeks. The participants could have one day a week on which they did not consume them. The compliance with consuming the intervention foods as instructed was assessed by records in which the participants reported their daily use of the intervention foods during the whole WM. The compliance was calculated as the actual use of the intervention foods as a percentage of the instructed use. In case the participant consumed the intervention foods more than instructed, the compliance could account for > 100%. The total dietary intake was assessed by 4-day food records collected five times during the study: before the VLED (baseline) and on 6th, 12th, 18th and 24th week from the beginning of the WM.

### Body composition and anthropometric measurements

Body weight, waist circumference, and body composition were measured after a 12-h fast (2 dl of water was allowed) in light clothing at study clinic visits. The measurements were made at baseline, at the beginning of the WM (week 0), and on 12th and 24th week from the beginning of the WM.  Body weight was measured using a digital scale (Vogel & Halke, Hamburg, Germany, sensitivity ± 0.1 kg).  Waist circumference was measured halfway between the lowest rib and the iliac crest. FM, body fat-free mass (FFM) and body fat percent were determined by bioelectrical impedance (STA/BIA Body Composition Analyzer, Akern Bioresearch Srl, Firenze, Italy). To enhance the reproducibility of the BIA measurements, the participants were instructed to refrain from vigorous exercise 24 h before and alcohol consumption 48 h before the measurements to minimize variation in fluid balance between the visits. All BIA measurements were performed by the same trained laboratory technician. Height was measured at baseline using a wall-mounted stadiometer. BMI was calculated by dividing weight (kg) by height squared (m^2^).

### Biochemical measurements

Blood samples for fasting leptin, insulin, ghrelin, PYY, and glucose were collected at baseline and on 0, 12th and 24th week of the WM at study clinic visits. The samples were collected in the morning after a 12-h fast (2 dl of water was allowed). Prechilled fluoride citrate-containing tubes were used for glucose and EDTA-containing tubes for insulin, ghrelin, and PYY. Tubes containing clot activator were used for leptin.

Plasma ghrelin and PYY samples were centrifuged within 15 min after being drawn at 1700×*g* at 4 °C for 15 min and plasma glucose and insulin samples at 2400×*g* at 4 °C for 10 min. Serum leptin samples were allowed to clot at room temperature for 30 min and were then centrifuged at 2400×*g* at room temperature for 10 min. The samples were frozen and stored at − 70 °C until assayed. The samples of each participant were analysed in duplicate.

Plasma glucose was analysed using an enzymatic photometric assay (Konelab 20XTi Clinical Chemistry Analyzer, Thermo Fisher Scientific, Vantaa, Finland). The intra-assay CV was 2.7% at 10.2 mmol/l and the inter-assay CV 4.1% at 2.05 mmol/l and 1.8% at 8.2 mmol/l. Plasma insulin was analysed using a chemiluminometric immunoassay (ADVIA Centaur Immunoassay System, Siemens Medical Solution Diagnostics, Tarrytown, NY, USA). The intra-assay CV was 2.7% at 96 mU/l and the inter-assay CV was 6.6% at 5.9 mU/l and 5.1% at 64 mU/l.

Total plasma ghrelin (i.e. acylated and deacylated ghrelin), total plasma PYY (i.e. PYY_1–36_ and PYY_3–36_) and serum leptin were analysed using radioimmunoassay (RIA) kits (Linco Research, St. Charles, Mo, USA). The intra-assay CV for the total ghrelin RIA kit was 9.5% at 506 pg/ml and 8.2% at 1220 pg/ml and the inter-assay CV was 8.1% at 644 pg/ml and 13.5% at 1580 pg/ml. The intra-assay CV for the total PYY RIA kit was 11% at 62 pg/ml and 8% at 212 pg/ml and the inter-assay CV was 11.3% at 58 pg/ml and 8.8% at 207 pg/ml. The intra-assay CV for serum leptin RIA kit was 5.9% at 3 ng/ml and 5.1% at 21.7 ng/ml and the inter-assay CV was 6.0% at 3 ng/ml and 5.7% at 21.7 ng/ml. All the analyses were conducted in the University of Eastern Finland, Institute of Public Health and Clinical Nutrition.

### Statistical analyses

The sample size was estimated based on the expectation that the HSF group would maintain a ≥ 5% lower body weight than the LSF group at the end of the WM (expected difference in body weight between the groups: 5.5 kg, standard deviation of body weight at the end of the WM: 8.5 kg). The sample size calculation was based on differences in body weight because it was the primary outcome variable of the intervention. Based on power calculations, 38 participants/group were required to provide 80% power to detect a between-group difference of 5% in body weight with a 5% type I error.

Statistical analyses were conducted using IBM SPSS Statistics version 25.0 software (IBM Statistics for Windows, Armonk, NY: IBM Corp.). Results are expressed as means and standard deviation unless otherwise stated. The normality of distribution of variables and residuals was assessed by Shapiro–Wilk test. One measurement of total PYY at week 24 was excluded from the analyses because of a value more than five standard deviations from the mean.

Changes in outcome variables between the given study weeks were calculated as follows:

$${\text{Change between study weeks}} = {\text{value at the latter study week}} - {\text{value at the former study week}}$$

Percentage changes in outcome variables during the WL and the whole study were calculated as follows:

$${\text{Percentage change during the WL or the whole study}} = \left[ {\left( {{\text{value at the end of the WL or at the end of the study}} - {\text{value at baseline}}} \right) \div {\text{value at baseline}}} \right] \times {1}00$$

Differences between the groups in the changes in dietary intake from baseline to the WM were tested using the independent samples *t* test (normally distributed variables) and Mann–Whitney test (non-normally distributed variables). Dietary intake during the WM was calculated as the mean of 4-day food records completed on weeks 6, 12, 18 and 24 of the WM. The intake at baseline was subtracted from the mean intake during the WM to obtain the changes in dietary intake.

To test the effect of the intervention and the differences between the groups in anthropometric and biochemical measurements during the intervention (baseline and 0, 12th, and 24th week of the WM), a linear mixed model analysis with the restricted maximum-likelihood method was utilised with the group, time and group x time interaction as fixed effects and participant as a random effect. If an overall main effect was significant, post hoc analyses were conducted for pairwise comparisons with the Bonferroni adjustment. Log transformation was made for measurements of insulin and PYY to obtain normally distributed residuals.

If there were no differences between the groups in given outcome variables, the data were pooled for multivariable linear regression analyses to explore the associations between the changes in hormone or glucose concentrations (dependent variable) and the changes in FM and FFM (independent variables) during the WM. The associations were investigated in two phases of the WM: (1) early phase (from week 0 to week 12 of the WM) and (2) late phase (from week 12 to week 24 of the WM). The changes in FM and FFM were entered into the model first (unadjusted model). The model was further adjusted for potential confounding variables, i.e. age, sex, study group and hormone/glucose concentration at the beginning of the corresponding phase of the WM (adjusted model).

## Results

### Participants

Eighty-two participants (21 males, 61 females) completed the study (completers) and were included in the current analyses. The mean age of the completers was 49.3 ± 9.3 years, body weight 95.2 ± 11.9 kg, and BMI 34.2 ± 2.5 kg/m^2^ at baseline. The groups did not differ in either the anthropometric or biochemical measurements at baseline (Table 2).

**Table 2:** Characteristics of the participants, anthropometric and biochemical measurements before the weight loss (baseline) and during the 24-week weight maintenance period

	Higher-satiety food group (*n* = 42)				Lower-satiety food group (*n* = 40)				*p*^a^		
	Baseline	0 wks WM	12 wks WM	24 wks WM	Baseline	0 wks WM	12 wks WM	24 wks WM	Group	Time	Group × time
Sex, male/female	12/30				9/31						
Age (years)	49.6 ± 9.5				49.1 ± 9.1						
Weight (kg)	95.7 ± 10.8	83.7 ± 8.9	83.5 ± 9.8	85.0 ± 10.7	94.6 ± 13.1	82.6 ± 10.2	82.4 ± 10.7	83.5 ± 11.0	0.599	< 0.001	0.928
BMI (kg/m^2^)	34.0 ± 2.3	29.8 ± 2.1	29.7 ± 2.3	30.2 ± 2.6	34.3 ± 2.7	30.0 ± 2.3	29.9 ± 2.6	30.3 ± 2.7	0.683	< 0.001	0.914
FM (kg)	37.8 ± 6.0	29.3 ± 5.8	28.7 ± 6.4	30.5 ± 7.0	38.1 ± 7.1	29.3 ± 6.4	28.4 ± 5.8	30.1 ± 6.5	0.997	< 0.001	0.863
FM (%)	39.6 ± 5.2	35.1 ± 5.8	34.2 ± 6.1	35.8 ± 6.4	40.5 ± 6.2	35.7 ± 6.7	34.7 ± 6.4	36.2 ± 6.5	0.608	< 0.001	0.969
FFM (kg)	57.9 ± 9.3	54.3 ± 7.8	55.0 ± 8.4	54.5 ± 8.4	56.6 ± 11.6	53.3 ± 9.7	54.0 ± 10.2	53.3 ± 10.3	0.565	< 0.001	0.952
WC (cm)	106.0 ± 10.2	93.9 ± 9.2	94.2 ± 9.8	95.0 ± 10.7	103.5 ± 9.5	92.6 ± 7.9	92.4 ± 8.5	93.2 ± 8.9	0.351	< 0.001	0.471
fP-Glucose (mmol/l)	6.2 ± 0.6	5.6 ± 0.5	5.6 ± 0.5	5.8 ± 0.6	6.0 ± 0.6	5.6 ± 0.5	5.6 ± 0.4	5.7 ± 0.4	0.646	< 0.001	0.531
fP-Insulin^b^ (mU/l)	15.0 ± 10.9	8.6 ± 6.5	9.1 ± 5.2	9.6 ± 5.4	14.2 ± 7.0	8.1 ± 4.6	8.9 ± 4.7	8.7 ± 4.7	0.690	< 0.001	0.545
fS-Leptin (ng/ml)	24.1 ± 9.7	9.7 ± 5.9	14.9 ± 8.0	17.0 ± 8.8	27.1 ± 10.7	11.2 ± 6.3	17.0 ± 10.2	18.6 ± 9.8	0.252	< 0.001	0.604
fP-Ghrelin (pg/ml)	839.6 ± 263.7	985.0 ± 329.5	902.3 ± 295.9	902.5 ± 289.7	939.1 ± 257.7	1082.1 ± 266.1	1024.3 ± 303.0	1017.3 ± 320.7	0.084	< 0.001	0.809
fP-PYY^b^ (pmol/l)	96.1 ± 33.7	84.8 ± 30.3	88.8 ± 27.8	89.8 ± 26.8	94.7 ± 29.3	87.8 ± 28.9	90.9 ± 27.1	94.4^c^ ± 29.8	0.605	0.001	0.801

Data are expressed as mean ± standard deviation

*wks* weeks, *WM* weight maintenance period, *FM* body fat mass, *FFM* body fat-free mass, *WC* waist circumference, *PYY* peptide YY

^a^The effect of group, time, and group x time interaction using linear mixed model analysis

^b^Log-transformed values used in the analysis

^c^*n* = 39

Compliance (i.e. the use of intervention foods in relation to the instructed use) did not differ between the HSF and LSF groups (100.8 ± 9.0% vs. 99.1 ± 9.1% of the instructed use, *p* = 0.33; HSF vs. LSF).

### Dietary intake in the study groups

At baseline, the percentage of total energy intake (E%) from carbohydrates was higher in the LSF than in the HSF group (Table 3). Otherwise, baseline dietary intake did not differ between the groups.

**Table 3:** Dietary intake before the weight loss (baseline) and the changes from baseline during the 24-week weight maintenance period assessed by 4-day dietary records

	Higher-satiety food group (*n* = 42)		Lower-satiety food group (*n* = 40)		*p*^b^
	Baseline	WM^a^—baseline	Baseline	WM^a^—baseline	
Energy (kcal/day)	2053.2 ± 526.4	− 329.8 ± 399.4	1945.4 ± 558.1	− 204.4 ± 450.2	0.185
Protein (g/day)	86.4 ± 20.7	+ 19.3 ± 16.4	79.4 ± 21.4	− 0.9 ± 18.5	< 0.001
Protein (E%)	17.1 ± 2.6	+ 8.3 ± 3.6	16.6 ± 2.5	+ 1.4 ± 2.4	< 0.001
Carbohydrates (g/day)	222.7 ± 62.2	− 37.1 ± 50.6	226.0 ± 62.0	− 13.4 ± 51.1	0.051
Carbohydrates (E%)	43.7 ± 7.3^c^	− 0.4 ± 5.8	47.2 ± 7.1^c^	0.0 ± 6.2	0.751
Fat (g/day)	71.3 ± 25.5	− 19.2 ± 15.4	69.2 ± 29.7	− 7.5 ± 27.4	0.002
Fat (E%)	31.2 ± 7.1	− 4.1 ± 6.1	31.5 ± 5.6	+ 1.1 ± 6.3	< 0.001
Dietary fibre (g/day)	22.8 ± 7.0	+ 9.7 ± 8.4	23.4 ± 9.5	− 0.1 ± 7.9	< 0.001

Data are expressed as mean ± standard deviation

*E%* percentage of total energy intake, *WM* weight maintenance period

^a^Dietary intake during the WM was calculated as the mean intake of weeks 6, 12, 18 and 24 of the WM

^b^The difference between the groups in the changes in dietary intake using independent samples *t* test (normally distributed variables) and Mann–Whitney test (non-normally distributed variables)

^c^The difference between the groups at baseline *p* = 0.03 (independent samples *t* test)

During the WM, total energy and carbohydrate intake decreased from baseline similarly in both groups. Intakes of protein and dietary fibre increased, and intake of fat decreased more in the HSF than in the LSF group (Table 3). Consequently, the mean (all time points during the WM combined) intakes of protein (25.5 ± 3.2 vs. 18.0 ± 1.7 E%, *p* < 0.001; HSF vs. LSF) and dietary fibre (32.6 ± 8.4 vs. 23.3 ± 7.0 g/day, *p* < 0.001) were higher whereas the intakes of fat (27.1 ± 3.9 vs. 32.6 ± 3.8 E%, *p* < 0.001) and carbohydrates (43.3 ± 4.6 vs. 47.2 ± 3.2 E%, *p* < 0.001) were lower in the HSF than in the LSF group. The mean energy intake during the WM was similar in the groups (1723 ± 379 vs. 1741 ± 380 kcal/day, *p* = 0.8; HSF vs. LSF).

### Anthropometric and biochemical measurements in the study groups

Within both groups, all the anthropometric measures decreased during the WL and remained below the baseline measurements at week 24 of the WM (Table 2). After the WL, fasting ghrelin concentrations had increased whereas fasting leptin, insulin, PYY, and glucose concentrations had decreased. These changes were retained in both groups during the WM, apart from that of PYY, which reverted to the baseline concentration (Table 2). However, there were no statistically significant group x time interactions in any of the anthropometric or biochemical measurements. Thus, detailed changes in these variables during the study were assessed in the whole study sample.

### Body weight and composition in the whole study sample

During the WL, weight loss was on average 12.0 ± 3.5 kg (*p* < 0.001). During the early phase of the WM (weeks 0–12 of the WM), mean body weight did not change. During the late phase of the WM (weeks 12–24 of the WM) body weight increased by 1.3 ± 1.9 kg (*p* = 0.004); resulting in a 11.4% (*p* < 0.001) reduction from the baseline body weight at the end of the WM.

**Fig. 2:**
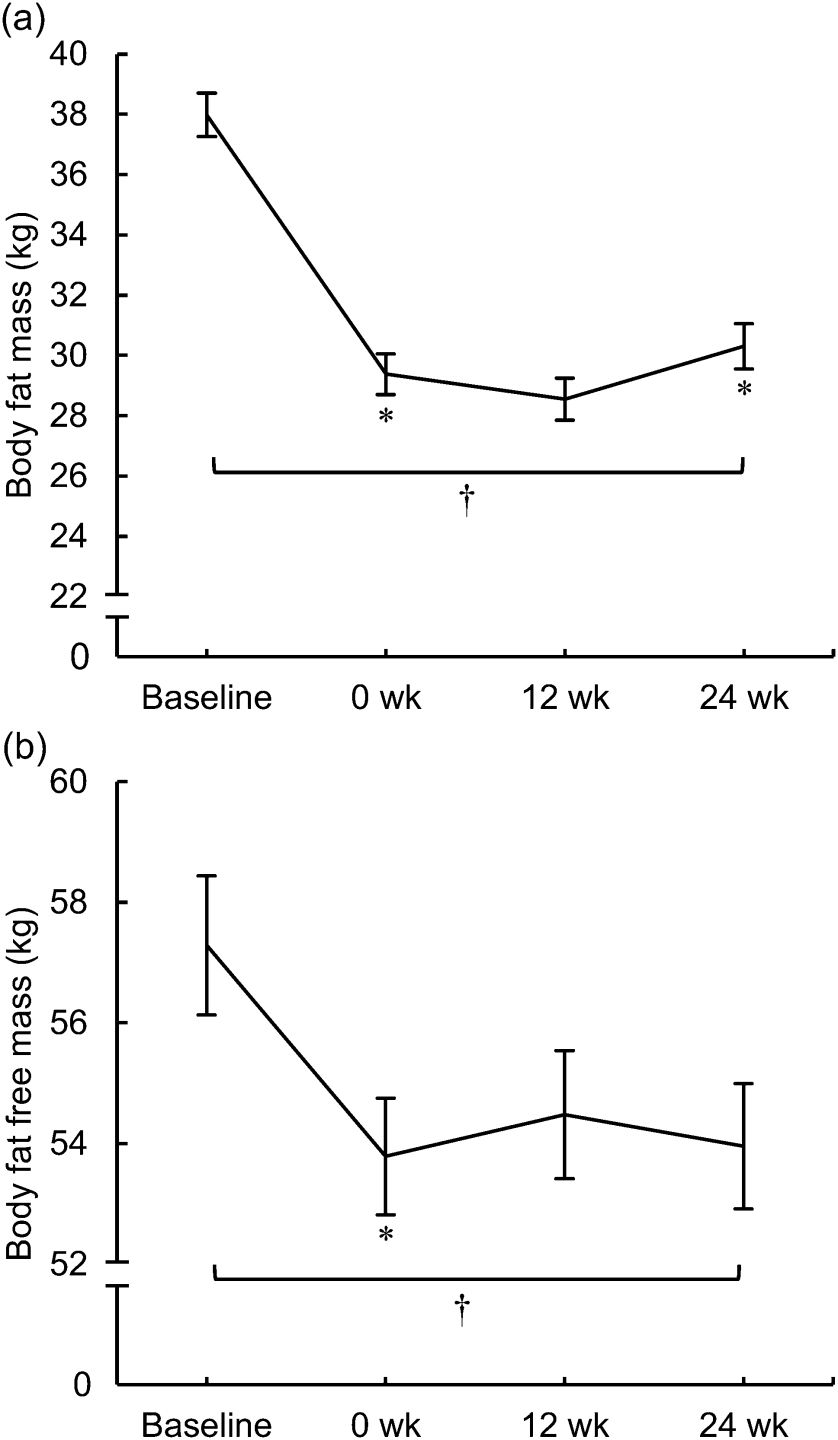
**a** Body fat mass and **b** body fat-free mass in the whole study sample (*n* = 76) before the very-low-energy diet (Baseline) and at 0, 12, and 24 weeks of the weight maintenance period. The values are means ± standard errors represented by vertical bars. *Mean value was significantly different from that of the value in the previous study week (*p* < 0.001). ^†^Mean value at week 24 was significantly different from that of the baseline value (*p* < 0.001). Significant differences between study weeks were determined via linear mixed model analysis and Bonferroni adjusted post hoc pairwise comparisons of a significant effect of time

During the WL, FM decreased by 8.6 ± 2.1 kg and FFM by 3.5 ± 2.5 kg (both: *p* < 0.001). FM increased during the late phase of the WM, whereas FFM did not change during either phase of the WM (Fig. 2a and b). At the end of the WM, FM was 20.2% and FFM 5.6% lower than at baseline (both: *p* < 0.001). There were extensive interindividual variations in the changes in FM and FFM (early phase of the WM: FM range − 4.7 to + 4.7 kg, FFM − 4.0 to + 5.3 kg; late phase of the WM: FM − 3.1 to + 4.8 kg, FFM − 5.5 to + 3.5 kg).

### Biochemical measurements in the whole study sample

**Fig. 3:**
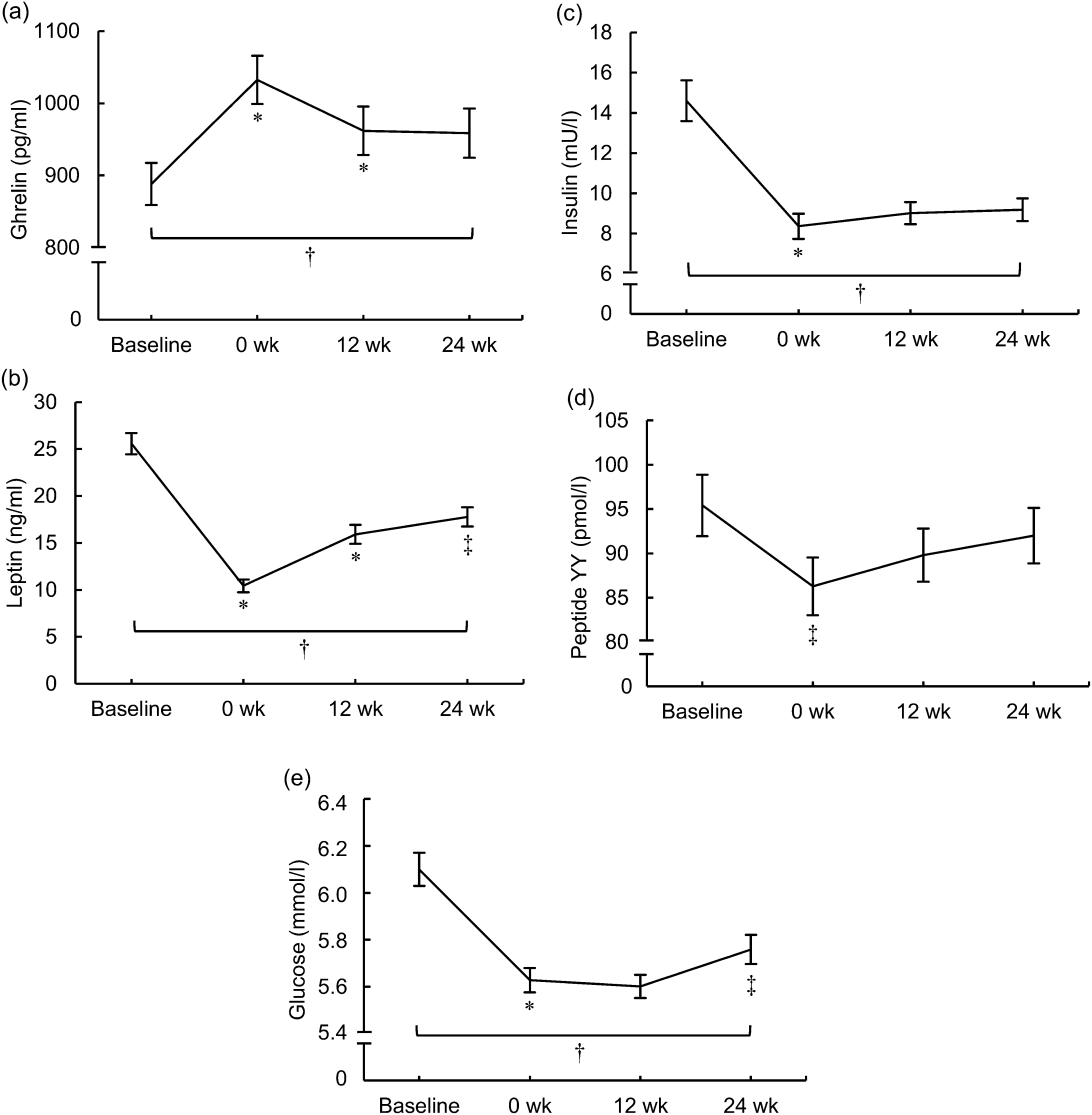
Concentrations of fasting **a** ghrelin, **b** leptin, **c** insulin, **d** peptide YY (PYY), and **e** glucose in the whole study sample (*n* = 82, for PYY *n* = 81) before the very-low-energy diet (baseline) and at 0, 12, and 24 weeks of the weight maintenance period. The values are means ± standard errors represented by vertical bars. *Mean value was significantly different from that of the value in the previous study week (*p* < 0.001). ^†^Mean value at week 24 was significantly different from that of the baseline value (*p* < 0.001). ^‡^Mean value was significantly different from that of the value in the previous study week (*p* < 0.05). Significant differences between study weeks were determined via linear mixed model analysis and Bonferroni adjusted post hoc pairwise comparisons of a significant effect of time. Log-transformed values were used in the analyses of insulin and PYY

After the WL, the mean concentration of fasting total ghrelin had increased (+ 17.4%, *p* < 0.001). During the early phase of the WM, ghrelin levels decreased (Fig. 3a). Thereafter, the decrease was attenuated; at the end of the WM, ghrelin levels remained 8.5% (*p* < 0.001) higher than at baseline.

During the WL, the mean fasting leptin concentration declined from baseline (− 60.3%, *p* < 0.001). In both phases of the WM, leptin levels increased (Fig. 3b) leading to 31.7% (*p* < 0.001) lower levels at the end of the WM compared to baseline.

The mean fasting insulin concentration decreased (− 38.4%, *p* < 0.001) during the WL and remained unchanged during both phases of the WM (Fig. 3c). At the end of the WM, insulin levels were 32.6% (*p* < 0.001) lower than at baseline.

During the WL, the mean fasting total PYY concentration decreased (− 6.7%, *p* = 0.001). In both phases of the WM, PYY levels increased, although not statistically significantly (*p* > 0.05), (Fig. 3d) causing PYY to revert to baseline levels at the end of the WM (*p* = 1.0).

The mean fasting glucose concentration decreased (− 7.3%, *p* < 0.001) during the WL and remained unchanged during the early phase of the WM (Fig. 3e). During the late phase of the WM, glucose levels increased but remained 5.3% (*p* < 0.001) lower than at baseline.

### Associations between the changes in hormone concentrations and body composition in the whole study sample

The changes in ghrelin concentrations were inversely associated with the changes in FFM during the early phase, but not the late phase of the WM (Table 4). No associations were found between the changes in ghrelin levels and FM in either phase of the WM.

**Table 4:** Associations between changes in body composition and in fasting hormone concentrations during the 24-week weight maintenance period in the whole study sample

	Change in ghrelin (pg/ml)			Change in leptin (ng/ml)			Change in insulin (mU/l)		
	*β*	95% CI	*p*	*β*	95% CI	*p*	*β*	95% CI	*p*
Weeks 0–12 of the WM (*n* = 76)									
Unadjusted model		*R*^2^ = 0.099			*R*^2^ = 0.037			*R*^2^ = 0.014	
Change in FM (kg)	− 6.62	− 20.60, 7.36	0.348	0.47	− 0.11, 1.04	0.108	0.18	− 0.25, 0.62	0.404
Change in FFM (kg)	− 24.77	− 42.21, − 7.33	0.006	0.06	− 0.66, 0.77	0.876	0.22	− 0.32, 0.77	0.419
Adjusted model		*R*^2^ = 0.202			*R*^2^ = 0.237			*R*^2^ = 0.333	
Change in FM (kg)	− 11.16	− 25.28, 2.95	0.119	0.68	0.14, 1.23	0.015	0.41	0.02, 0.79	0.038
Change in FFM (kg)	− 28.54	− 45.91, − 11.16	0.002	0.22	− 0.46, 0.90	0.520	0.32	− 0.16, 0.79	0.187
Weeks 12–24 of the WM (*n* = 75)									
Unadjusted model		*R*^2^ = 0.205			*R*^2^ = 0.194			*R*^2^ = 0.044	
Change in FM (kg)	− 17.19	− 36.45, 2.07	0.079	1.11	0.57, 1.65	< 0.001	0.47	− 0.06, 0.99	0.080
Change in FFM (kg)	− 6.63	− 27.31, 14.04	0.525	0.24	− 0.34, 0.81	0.419	0.07	− 0.49, 0.63	0.795
Adjusted model		*R*^2^ = 0.106			*R*^2^ = 0.337			*R*^2^ = 0.216	
Change in FM (kg)	− 17.15	− 36.85, 2.55	0.087	1.48	0.94, 2.02	< 0.001	0.59	0.09, 1.09	0.022
Change in FFM (kg)	− 5.14	− 26.39, 16.11	0.631	0.57	− 0.004, 1.14	0.051	0.25	− 0.29, 0.79	0.359

Values are unstandardised *β*-coefficients with corresponding 95% Confidence Intervals (CI) and *p* values obtained from multivariable linear regression analysis. The adjusted models were adjusted for age, sex, study group and hormone concentration at the beginning of the corresponding WM phase. Changes were calculated by subtracting the measurement at the beginning of the WM phase from the measurement at the end of the WM phase

*WM* weight maintenance period, *FM* body fat mass, *FFM* body fat-free mass

In the early phase of the WM, changes in leptin concentrations were associated with FM changes in the adjusted model (covariates: age, sex, study group, leptin concentration at week 0 of the WM), but not in the unadjusted model (Table 4). In the late phase of the WM, this association was found in both regression models.

In the early and late WM phases, changes in insulin concentrations were associated with FM changes in the adjusted model (covariates: age, sex, study group, insulin concentration at week 0 (early phase) or 12 (late phase) of the WM), but not in the unadjusted model (Table 4).

The changes in PYY or glucose concentrations were not associated with the changes in body composition during any phase of the WM in either of the regression models (data not shown).

## Discussion

In the present study, we found that weight loss increased fasting total plasma ghrelin concentrations and decreased fasting serum leptin and plasma insulin and glucose concentrations. These changes, as well as the weight reduction, were maintained for 6 months after the weight loss. In contrast, the fasting total plasma PYY concentrations returned to pre-weight loss levels. Interestingly, we found no differences in these changes between the groups consuming isoenergetic weight maintenance diets with different predetermined satiety values and macronutrient compositions, mainly consisting of differences in protein (26 vs. 18 E%) and dietary fibre (33 vs. 23 g/day intake. Dietary factors, such as dietary fibre and protein, modulate endogenous and gut microbial metabolisms [22], which may influence some hormones and glucose levels during a subsequent meal [23] and in a fasted state [24]. Diets with different macronutrient compositions resulted in different microbial-derived fasting serum short-chain fatty acid profiles which were related to fasting leptin and ghrelin levels [25]. Thus, we anticipated the two diets to induce different fasting appetite-related hormone levels. However, we found that in the weight-reduced participants, the protein and dietary fibre content of an isoenergetic weight maintenance diet had no effect on the fasting concentrations of glucose or on those of the appetite-related hormones ghrelin, leptin, insulin, and PYY.

Similar to our results, no differences were found in fasting leptin or insulin levels during a 12-week WM after VLED between two groups consuming isoenergetic diets with divergent protein (35 vs. 16 E%) and carbohydrate (42 vs. 63 E%) contents [13]. In a crossover study, a 4-week WM diet after a  12-week WL with 30 E% of protein and 10 E% of carbohydrates had similar effects on fasting ghrelin and PYY concentrations as isoenergetic diets containing 20 E% of protein and 60 E% or 40 E% of carbohydrates [16]. However, fasting insulin [16] and leptin [15] levels were lower after the diet with 30 E% of protein than after the other two diets, although the weight changes were comparable between the diets. Difference in the carbohydrate intake between the diets was large (from 20 to 50 E%) as compared to that of our study (4 E%). Thus, higher protein to carbohydrate ratio of a weight maintenance diet seemed to attenuate post-weight loss secretion of adiposity signals, leptin and insulin but did not affect PYY and ghrelin secretion. In these studies [15, 16], the participants were on average 20 years younger than those of our study, which may explain, at least in part, the finding of a blunting effect of higher-protein diet on leptin and insulin levels, which was undetected in our study. Increasing age, independently of weight status, may have inverse relationship with basal leptin secretion [26] and responsiveness in basal insulin release [27].

In addition, other results suggest that quite large differences in the carbohydrate or dietary fibre intakes are needed to induce changes in post-weight loss appetite-related hormone concentrations [14, 17]. Sloth et al. [14] compared three isoenergetic WM diets with differing carbohydrate (43 vs. 57 vs. 50 E%) and dietary fibre (39 vs. 36 vs. 28 g/day) contents but similar protein intakes (15 vs. 16 vs. 16 E%). They found that there was a greater increase in fasting insulin levels during the WM in the group with the lowest dietary fibre intake. In contrast, fasting PYY or glucose concentrations did not differ between the groups. Fermentation products of dietary fibre produced by gut microbiota might have an overnight-lasting effect on fasting insulin levels [28]. Recently, Ebbeling et al. [17] reported a greater decrease in the fasting ghrelin concentrations and a smaller increase in the fasting leptin concentrations during a 20-week WM in a low-carbohydrate (20 E%) diet group than in high- (60 E%) and moderate- (40 E%) carbohydrate diet groups. Protein intake was standardised (20 E%) across the groups, and all groups maintained a 10% weight loss. These results contradict the findings that a higher protein to carbohydrate ratio was the dietary determinant of basal hormonal levels after weight loss [15, 16]. Currently, the role of protein in the context of divergent dietary fibre and carbohydrate contents of WM diets remains unclear.

We found that during the first months of the WM, fasting ghrelin concentrations decreased as FFM slightly increased. Previously, weight loss-induced changes in ghrelin concentrations in subjects with overweight or obesity have been associated with changes in FFM but not in FM [29]. The same observation has been made in people with normal body weights [30]. Mechanisms that underlie the association between post-weight loss changes in FFM and ghrelin are unclear. However, ghrelin might have widespread physiological effects via different, partly unidentified, subtypes of the growth hormone secretagogue-receptor in endocrine and non-endocrine tissues including muscle [31]. In in-vitro and in animal models, ghrelin (either its acylated or deacylated form) enhanced muscle cell differentiation and regeneration [32, 33] and sustained muscle cell metabolism [34]. Although FFM comprehends all lean parts of the body, the decline in ghrelin levels concomitantly with FFM regain may be the result of indirect negative feedback mechanisms related to muscle cell function after weight loss. However, we found that during the late phase of the WM, the decrease in ghrelin levels became attenuated and the association with FFM disappeared. At the end of the study, ghrelin levels remained elevated compared to the levels before weight loss. This may indicate a disruption in the relationship between ghrelin and body mass during prolonged maintenance of reduced weight. Previously, elevated ghrelin levels were observed 6 months [35] and 12 months [7] after weight loss regardless of the tendency for weight regain and 12 months after weight loss with no weight regain [8, 36]. These results suggest that 6–12 months after weight loss ghrelin secretion may not have adjusted to the reduced body weight. However, the reasons for this possible maladaptation remains unclear. Nonetheless, the finding may partly explain the previous finding of increased subjective hunger feelings that sustained for 12 months after WL [7, 36].

Finding of an inverse association between the changes in ghrelin concentrations and FFM, but not FM, during the early phase of the WM is interesting. Fasting ghrelin levels were increased after short-term fasting and weight loss [4, 37], indicating that ghrelin has a role in meal initiation and regulation of energy balance. Our observation may shed new insight to the current view which suggests that this regulation occurs via an interaction between adipose tissue and peripheral hormones, thus emphasising the central role of FM in the control of food intake [38, 39]. Higher ghrelin levels have been associated with lower resting metabolic rate and thermogenic effect of food in women with normal body weights [40]. Moreover, blocking ghrelin signalling in rat brain regions involved in energy expenditure regulation resulted in decreased  body weight compared to controls without affecting food intake, demonstrating a central mechanism of ghrelin action on energy expenditure [41]. In contrast, FFM is the primary contributor of resting [42] and basal [43] metabolic rates which determine about 70% of daily energy requirements. Furthermore, Blundell et al. [44] observed that FFM, and not FM, was associated with energy intake in subjects with obesity. Dulloo et al. [45] have presented a theory of collateral fattening, i.e. the reduction of FFM is the primary contributor to weight regain after weight loss. This leads to an excessive increase in FM. The previous and our observations suggest that FFM affects food intake, and the post-weight loss FFM regain relates to lowering of ghrelin levels, which, in turn, may contribute to higher energy expenditure. Moreover, this finding is important since it may indicate that sparing FFM during obesity treatment involving energy restriction might attenuate the weight loss-induced increase in ghrelin levels.

In this study, the post-weight loss changes in leptin and insulin levels were associated with changes in FM independently of potential confounders, e.g. age and sex. Fasting leptin and insulin levels remained even lower than before the weight loss. This indicates that after stabilisation of weight reduction, there are concurrent changes in leptin and insulin secretion and FM. This reflects the normal fluctuation of insulin and leptin levels according to the energy balance. Fasting leptin and insulin concentrations were reported to remain reduced for 12 months after weight loss [8, 36], also after a partial regain of body weight [5, 7]. However, similarly to our results with FM, Sumithran et al. [7] found that the increase in leptin levels during the WM correlated with the body weight regain. Another study found that changes in insulin concentrations and body weight were related when the whole study period (i.e. WL and WM) was examined [36]. Furthermore, leptin levels remained decreased in those who succeeded in maintaining their reduced weight but not in those who regained weight [46]. Our results are in line with these previous observations, suggesting that leptin and insulin secretion adapts to the body’s reduced energy reserves during the WM.

In our intervention, we found that fasting PYY levels declined during energy restriction. However, PYY returned to its pre-weight loss concentrations during the WM. This confirms recent findings of a 12-month WM study after weight reduction [8]. In contrast, the PYY levels remained decreased during a 1-year WM regardless of a 50% regain of lost body weight after a VLED-induced weight loss [7]. In another study, fasting PYY concentrations increased during an 8% weight loss achieved in 6 months and during weight stabilisation thereafter [35]. The differences in the magnitude and rate of weight loss as well as the significant variation in PYY concentrations in different studies may contribute to these divergent observations.

The strength of our study was the lack of differences between the groups in energy intake and in changes in body weight, which enabled us to investigate the effect of the macronutrient content of the diet on hormone secretion without any confounding by differences in energy intakes or weight changes. In addition, measurements were taken three times during the WM. Despite these strengths, there are certain limitations to be considered. We measured only fasting concentrations of hormones and glucose. Many of the investigated signals affect hunger and satiety postprandially [12]. We did not investigate the effect of WM diet on postprandial secretion, and thereby on meal-induced satiety. However, leptin, insulin, and ghrelin are recognised as tonic signals from the periphery, reflecting energy balance and interacting with gut-derived satiety signals [37]. Thus, alterations of basal levels of these signals have satiety-related effects. We did not measure subjective hunger and satiety feelings but focus to investigate whether isoenergetic diets with differing macronutrient contents and with similar effects on body weight could affect fasting appetite-related hormone concentrations. Moreover, we did not want to emphasise the subjective experience of appetite to minimise the influence of participants’ explicit evaluations on food intake. Instead, the aim was to let hunger and satiety feelings initiate food intake at a more unconscious level. Sample size was determined based on calculated power to detected differences in body weight instead of hormonal outcomes. The sample size in our study was, however, relatively large compared to previous studies with comparable designs investigating appetite-related hormones [13, 14, 18]. We assessed total ghrelin and total PYY and not the biologically active forms (acylated ghrelin and PYY_3–36_, respectively). However, acylated ghrelin has been found to correlate with the total ghrelin concentration [47]. We used bioelectrical impedance to measure the body composition of the participants. The method is sensitive to different factors, e.g. fluid balance of the subject and recent physical activity, which may affect the accuracy of the measurements. However, we aimed to control these factors by giving the participants careful instructions about exercising and maintaining a similar fluid balance prior to the measurements. The assessment of dietary intake was made by self-reported food diaries, which may have introduced reporting bias to the data. In case this bias occurred, it may have been balanced across the groups not affecting the comparison of the groups.

In conclusion, our study demonstrated that in conditions of maintained weight loss, the macronutrient content of the isoenergetic weight maintenance diet did not affect post-weight loss changes in the fasting concentrations of appetite-related hormones. The weight loss-induced increase in the fasting ghrelin concentrations and the decrease in the fasting leptin and insulin levels, but not PYY concentrations, were still evident 6 months after the weight loss. However, leptin and insulin concentrations seemed to adjust to reduced FM and thus reach new homeostatic levels with the maintenance of reduced weight. After the weight loss, ghrelin concentrations seemed to decline in relation to the increase in FFM. This association between ghrelin secretion and FFM in the context of weight management warrants further investigation.

## Data Availability

Upon request from the corresponding author.

## References

[1] Berthoud HR, Münzberg H, Morrison CD (2017). Blaming the brain for obesity: integration of hedonic and homeostatic mechanisms. Gastroenterology.

[2] Batterham RL, Cohen MA, Ellis SM (2003). Inhibition of food intake in obese subjects by peptide YY_3–36_. N Engl J Med.

[3] Melby CL, Paris HL, Foright RM, Peth J (2017). Attenuating the biologic drive for weight regain following weight loss: must what goes down always go back up?. Nutrients.

[4] Cummings DE, Weigle DS, Frayo RS (2002). Plasma ghrelin levels after diet-induced weight loss or gastric bypass surgery. N Engl J Med.

[5] Lien LF, Haqq AM, Arlotto M (2009). The STEDMAN project: biophysical, biochemical and metabolic effects of a behavioral weight loss intervention during weight loss, maintenance, and regain. OMICS.

[6] Beck EJ, Tapsell LC, Batterham MJ, Tosh SM, Huang XF (2010). Oat beta-glucan supplementation does not enhance the effectiveness of an energy-restricted diet in overweight women. Br J Nutr.

[7] Sumithran P, Prendergast LA, Delbridge E (2011). Long-term persistence of hormonal adaptations to weight loss. N Engl J Med.

[8] Iepsen EW, Lundgren J, Holst JJ, Madsbad S, Torekov SS (2016). Successful weight loss maintenance includes long-term increased meal responses of GLP-1 and PYY_3-36_. Eur J Endocrinol.

[9] Leibel RL, Rosenbaum M, Hirsch J (1995). Changes in energy expenditure resulting from altered body weight. N Engl J Med.

[10] MacLean PS, Bergouignan A, Cornier MA, Jackman MR (2011). Biology’s response to dieting: the impetus for weight regain. Am J Physiol Integr Comp Physiol.

[11] Westerterp-Plantenga MS, Lemmens SG, Westerterp KR (2012). Dietary protein—its role in satiety, energetics, weight loss and health. Br J Nutr.

[12] Karhunen LJ, Juvonen KR, Huotari A, Purhonen AK, Herzig KH (2008). Effect of protein, fat, carbohydrate and fibre on gastrointestinal peptide release in humans. Regul Pept.

[13] Claessens M, van Baak MA, Monsheimer S, Saris WHM (2009). The effect of a low-fat, high-protein or high-carbohydrate ad libitum diet on weight loss maintenance and metabolic risk factors. Int J Obes.

[14] Sloth B, Due A, Larsen TM, Holst JJ, Heding A, Astrup A (2009). The effect of a high-MUFA, low-glycaemic index diet and a low-fat diet on appetite and glucose metabolism during a 6-month weight maintenance period. Br J Nutr.

[15] Ebbeling CB, Swain JF, Feldman HA (2012). Effects of dietary composition on energy expenditure during weight-loss maintenance. JAMA.

[16] Hron BM, Ebbeling CB, Feldman HA, Ludwig DS (2017). Hepatic, adipocyte, enteric and pancreatic hormones: response to dietary macronutrient composition and relationship with metabolism. Nutr Metab.

[17] Ebbeling CB, Feldman HA, Klein GL (2018). Effects of a low carbohydrate diet on energy expenditure during weight loss maintenance: randomized trial. BMJ.

[18] Garcia JM, Iyer D, Poston WSC (2006). Rise of plasma ghrelin with weight loss is not sustained during weight maintenance. Obesity.

[19] Heinonen MV, Laaksonen DE, Karhu T (2009). Effect of diet-induced weight loss on plasma apelin and cytokine levels in individuals with the metabolic syndrome. Nutr Metab Cardiovasc Dis.

[20] Crujeiras AB, Goyenechea E, Abete I (2010). Weight regain after a diet-induced loss is predicted by higher baseline leptin and lower ghrelin plasma levels. J Clin Endocrinol Metab.

[21] Karhunen L, Lyly M, Lapveteläinen A (2012). Psychobehavioural factors are more strongly associated with successful weight management than predetermined satiety effect or other characteristics of diet. J Obes.

[22] Wellington N, Shanmuganathan M, de Souza RJ (2019). Metabolic trajectories following contrasting prudent and western diets from food provisions: identifying robust biomarkers of short-term changes in habitual diet. Nutrients.

[23] Brighenti F, Benini L, Del Rio D (2006). Colonic fermentation of indigestible carbohydrates contributes to the second-meal effect. Am J Clin Nutr.

[24] Robertson MD, Henderson RA, Vist GE, Rumsey RD (2002). Extended effects of evening meal carbohydrate-to-fat ratio on fasting and postprandial substrate metabolism. Am J Clin Nutr.

[25] Mueller NT, Zhang M, Juraschek SP, Miller ER, Appel LJ (2020). Effects of high-fiber diets enriched with carbohydrate, protein, or unsaturated fat on circulating short chain fatty acids: results from the OmniHeart randomized trial. Am J Clin Nutr.

[26] Isidori AM, Strollo F, Morè M (2000). Leptin and aging: correlation with endocrine changes in male and female healthy adult populations of different body weights. J Clin Endocrinol Metab.

[27] Iozzo P, Beck-Nielsen H, Laakso M (1999). Independent influence of age on basal insulin secretion in nondiabetic humans. J Clin Endocirnol Metab.

[28] Boll EVJ, Ekström LMNK, Courtin CM (2016). Effects of wheat bran extract rich in arabinoxylan oligosaccharides and resistant starch on overnight glucose tolerance and markers of gut fermentation in healthy young adults. Eur J Nutr.

[29] Purnell JQ, Cummings D, Weigle DS (2007). Changes in 24-h area-under-the-curve ghrelin values following diet-induced weight loss are associated with loss of fat-free mass, but not with changes in fat mass, insulin levels or insulin sensitivity. Int J Obes.

[30] Scheid JL, De Souza MJ, Leidy HJ, Williams NI (2011). Ghrelin but not peptide YY is related to change in body weight and energy availability. Med Sci Sports Exerc.

[31] Gnanapavan S, Kola B, Bustin SA (2002). The tissue distribution of the mRNA of ghrelin and subtypes of its receptor, GHS-R, in humans. J Clin Endocrinol Metab.

[32] Filigheddu N, Gnocchi VF, Coscia M (2007). Ghrelin and des-acyl ghrelin promote differentiation and fusion of C2C12 skeletal muscle cells. Mol Biol Cell.

[33] Reano S, Angelino E, Ferrara M (2017). Unacylated ghrelin enhances satellite cell function and relieves the dystrophic phenotype in duchenne muscular dystrophy mdx model. Stem Cells.

[34] Han L, Li J, Chen Y, Wang W, Zhang D, Liu G (2015). Effects of ghrelin on triglyceride accumulation and glucose uptake in primary cultured rat myoblasts under palmitic acid-induced high fat conditions. Int J Endocrinol.

[35] Hill BR, Rolls BJ, Roe LS, De Souza MJ, Williams NI (2013). Ghrelin and peptide YY increase with weight loss during a 12-month intervention to reduce dietary energy density in obese women. Peptides.

[36] Nymo S, Coutinho SR, Eknes PH (2018). Investigation of the long-term sustainability of changes in appetite after weight loss. Int J Obes.

[37] Cummings DE (2006). Ghrelin and the short- and long-term regulation of appetite and body weight. Physiol Behav.

[38] Woods SC (2009). The control of food intake: behavioral versus molecular perspectives. Cell Metab.

[39] Sondergaard E, Gormsen LC, Nellemann B, Vestergaard ET, Christiansen JS, Nielsen S (2009). Visceral fat mass is a strong predictor of circulating ghrelin levels in premenopausal women. Eur J Endocrinol.

[40] St-Pierre DH, Karelis AD, Cianflone K (2004). Relationship between ghrelin and energy expenditure in healthy young women. J Clin Endocrinol Metab.

[41] Shrestha YB, Wickwire K, Giraudo S (2009). Effect of reducing hypothalamic ghrelin receptor gene expression on energy balance. Peptides.

[42] Ravussin E, Lillioja S, Anderson TE, Christin L, Bogardus C (1986). Determinants of 24-hour energy expenditure in man. Methods and results using a respiratory chamber. J Clin Invest.

[43] Johnstone AM, Murison SD, Duncan JS, Rance KA, Speakman JR (2005). Factors influencing variation in basal metabolic rate include fat-free mass, fat mass, age, and circulating thyroxine but not sex, circulating leptin, or triiodothyronine. Am J Clin Nutr.

[44] Blundell JE, Caudwell P, Gibbons C (2012). Body composition and appetite: fat-free mass (but not fat mass or BMI) is positively associated with self-determined meal size and daily energy intake in humans. Br J Nutr.

[45] Dulloo AG, Miles-Chan JL, Schutz Y (2018). Collateral fattening in body composition autoregulation: its determinants and significance for obesity predisposition. Eur J Clin Nutr.

[46] Mavri A, Stegnar M, Sabovic M (2001). Do baseline serum leptin levels predict weight regain after dieting in obese women?. Diabetes Obes Metab.

[47] Foster-Schubert KE, Overduin J, Prudom CE (2008). Acyl and total ghrelin are suppressed strongly by ingested proteins, weakly by lipids, and biphasically by carbohydrates. J Clin Endocrinol Metab.

